# The clinical effects of probiotics for inflammatory bowel disease: A meta-analysis: Erratum

**DOI:** 10.1097/MD.0000000000014429

**Published:** 2019-02-01

In the article, “The clinical effects of probiotics for inflammatory bowel disease: A meta-analysis”^[1]^ which appears in Volume 97, Issue 51 of *Medicine*, there is a correction on the official term of the drug name used in the article.

The drug term VSL#3 is being replaced with the generic name of the formulation “De Simone Formulation” following a legal dispute between the distributors of the product VSL#3 and the formula inventor.

This change does not affect the study of the paper.
